# Effects of Participation in Social Activities on Cognitive Function Among Middle-Aged and Older Adults in Korea

**DOI:** 10.3390/ijerph15102315

**Published:** 2018-10-21

**Authors:** Jongnam Hwang, Sangmin Park, Sujin Kim

**Affiliations:** 1 Division of Social Welfare and Health Administration, Wonkwang University, Iksan 54538, Korea; jonhwang416@gmail.com; 2 Department of Family Medicine & Biomedical Sciences, Seoul National University, Seoul 03080, Korea; smpark.snuh@gmail.com; 3 Department of Health Care Policy Research, Korea Institute for Health and Social Affairs, Sejong 30147, Korea

**Keywords:** cognitive function, social activity, older adults, Korea

## Abstract

Cognitive function is a critical health issue in later life, the decline of which disrupts well-being and daily life function. Cognitive decline in older ages can also be understood in the context of the social environment such as social connectedness and engagement in personal life. This study aimed to examine: (1) whether participation in social activities contributes to preventing cognitive decline, and (2) what type of social activities are beneficial to maintaining cognitive function. Data from the Korean Longitudinal Study of Aging (KLOSA) 2006–2014, a longitudinal survey of the household-dwelling population aged 45 and older in Korea were used. The results revealed that Mini-Mental State Examination (MMSE) scores decreased with increasing age, at a rate of approximately 0.18 units across all age-gender groups, and the decrease was steeper for adults aged 65 and over. Participation in social gatherings was likely to delay the decline in cognitive function after the age of 65. In a gender-stratified model, social activity may not have an impact on the decline of cognitive function for men, whereas participation in social gatherings was negatively related to the decline of MMSE scores in women. This study suggests the need for a gender-stratified policy for preventing the decline of cognitive function while promoting engagement in social activities in Korean older adults.

## 1. Introduction

Cognitive function is a critical health issue in later life [1,2], and the decline of which disrupts well-being and daily life function [3]. Accumulative studies have documented age-related decrements in cognitive function, indicating that cognitive decline is a general part of brain aging [4,5]. However, biological aging is not entirely responsible for cognitive impairment among older adults. Cognitive function in older ages can also be understood in the context of their social environment, such as social connectedness and engagement in personal life [2,6,7]. A review of the association between cognitive function and social lifestyle reported that social relationships, including engagement, activity, and networking, exhibit a close relationship with cognitive function [8,9]. Individuals who are less socially active are more likely to experience cognitive declines compared with those who lead more active social lifestyles [10]. These findings suggest that more social engagement in later life may help delay or prevent cognitive decline [11]. In fact, a burgeoning body of literature highlights the fact that greater participation in a wide range of social activities in later life contributes to the prevention of cognitive impairment [12]. It has been suggested that active participation in various activities may protect cognitive function by providing stimulation. A number of studies have reported that engaging in social activities is beneficial to maintaining better physical and mental health outcomes, including better cognitive function, ultimately leading to a reduced risk of dementia, particularly among older adults [13,14]. Participation in social activities offers important social roles, self-esteem, and social competence, which may protect against neuropathology (i.e., reducing the stress response) [15,16,17]. For instance, a community-based prospective study suggested that participation in senior citizen clubs or senior centers may produce beneficial effects in reducing cognitive decline in later life [18]. In studies of older American and Swedish adults, participating in social and leisure activities helped prevent cognitive decline [19,20]. Furthermore, a recent population-based prospective study reported that emotional exchanges in social situations and satisfaction in relationships are both protective factors against cognitive decline among older adults [21].

Participation in social activities is part of a socially-oriented sharing of individual resources, which includes a wide range of activities involving interactions with family, friends, and other social groups [22]. It has been suggested that social activities related to economic security and spiritual well-being may contribute to improving, or at least maintaining, physical and mental health, ultimately associated with longevity in older adults [11]. With respect to cognitive function, it is hypothesized that engagement with cognitively stimulating activity benefits its function, reducing the possibility of cognitive decline [23]. However, social activities do not have a consistently positive impact if participation in social groups brings about interpersonal conflict, which may lead to psychological distress, adversely impacting health [24,25]. In this sense, it could be understood that various social activities differently affect cognitive function, given that each activity has its own purpose. In addition to the potentially differential impacts of various activities, the benefits of social activities may differ by gender. For example, one cross-sectional study among Chinese older adults demonstrated that volunteer work was associated with better cognitive function among only women, whereas participating in hobby groups was associated with better cognitive function among both men and women [8]. Other studies suggested that older women might have more beneficial effects from involvement in social activity compared with men, because older men are more likely to receive social and emotional support from their spouses [18,26,27,28]. Thus, older women tend to gain noteworthy caring support from other social network, such as friends, colleagues, and neighborhoods, rather than their spouses [18,26,27,28].

Prevention, or at least the delay, of cognitive decline in later life is a prominent issue in the field of health policy. Although an increasing number of studies seek to understand the relationship between cognitive function and participation in social activities [6,11,13,29], it is still not clear whether different social activities have different impacts on cognitive function among middle-aged and older individuals in addition to different impacts based on gender, particularly the Korean population. Recent studies using data from the Korean Longitudinal Study of Aging (KLOSA) attempted to assess the relationship between different types of social activities and cognitive function in older adults, indicating that the role of social activity should receive more attention given the close association between participation in social activity and cognitive function [17,29]. Although their results may provide some insight into the relationship, these studies failed to accurately scrutinize the impact of social activities on cognitive function and overlooked gender differences in the relationship between social activity and cognitive decline. This could have been because of the study design, whether it was a cross-sectional or a comparison of certain points. To reduce the knowledge gap, this study aimed to examine: (1) whether participation in social activities contributes to preventing cognitive decline in older adults, and (2) what type of social activities are beneficial to maintaining cognitive function at older ages using nationally representative longitudinal survey data.

## 2. Materials and Methods

### 2.1. Study Population

Data were derived from the KLOSA, which was conducted by the Korean Employment Information Service. The KLOSA is a nationally representative longitudinal survey of the household-dwelling population aged 45 and older in Korea using multi-stage, stratified probability sampling [30]. A total of 10,254 individuals completed interviews at baseline (2006). Interviews were conducted every even-numbered year up to 2014, which resulted in five survey waves. At each wave, information regarding education, income, assets, employment, lifestyle, health status, and cognitive function was collected. The follow-up rate for each wave was 86.6%, 80.3%, 76.2%, and 72.8% for the 2008, 2010, 2012, and 2014 surveys, respectively [30]. The current study selected 7299 adults with normal cognitive function at baseline, which was defined as a Korean version of the Mini-Mental State Examination (K-MMSE) score ≥24. Given missing covariates at baseline, 593 participants were excluded from the study. Of 6706 eligible respondents, 5948 (88.7%) provided at least one memory score and 4453 (66.4%) provided four memory scores at all assessments during the follow-up period (after the baseline survey).

### 2.2. Assessment of Cognitive Function

Cognitive function measured by K-MMSE scores was used as a dependent variable. The K-MMSE, a structured questionnaire to measure global cognitive performance and screen for dementia, consists of 11 items in seven categories including orientation for time, place, and person, registration of three objects, attention and calculation, recall of three words, language, and visual construction [31]. Total K-MMSE scores range from 0 to 30, with normal cognitive function defined as score of 24 or greater [31,32]. The K-MMSE was validated elsewhere [32]. The present study used K-MMSE scores over the eight-year follow-up as outcome measures.

### 2.3. Assessment of Social Activity

Social activity was assessed at baseline using four questions: (1) “Do you participate in religious meetings?” (Religious parties), (2) “Do you participate in social clubs (Private savings clubs, senior citizens’ clubs), or leisure, cultural, or sports groups?” (Social gatherings), (3) “Do you participate in alumni societies, societies for people from the same hometown, or family councils?” (Alumni/clan gatherings), and (4) “Do you participate in volunteer groups, political parties, non-government organizations, or interest groups?” (Volunteer work). This study assessed the relationship between each social activity and cognitive performance.

### 2.4. Assessment of Covariates

Sociodemographic covariates included gender (male/female), age, educational status (elementary school, middle school, high school, or college and above), household income quartile (first quartile as the poorest), working status (yes/no), marital status (single/married), and residence (urban/rural). Based on the self-reported information, health-related covariates included physical activity, smoking status (ever/never smoked), alcohol use (non-drinkers/current drinkers), limited activities of daily living (0, ≥1), depression (10-item Center for Epidemiologic Studies–Depression Scale ≥4, <4), and comorbidity (at least one of hypertension, diabetes, cardiovascular disease, and/or cerebrovascular disease, 0). All covariates mentioned above were measured at baseline (2006).

### 2.5. Statistical Analysis

We used multi-level mixed models to test the hypothesis that individuals with higher baseline social activity would experience a slower rate of decline in cognitive performance during the follow-up period. The multi-level models allowed us to examine the trajectory of cognitive performance based on the level of baseline social activity, the average rate of cognitive performance over time.

First, to assess the overall change of K-MMSE scores related to increasing age, the difference between actual age and an age of 45 (age-45) was included. However, even if baseline social activity did not relate to the change of cognitive function in middle age, it could affect the change of cognitive function in old age (older than 65). Therefore, a spline at the age of 64 was introduced to assess the change of K-MMSE scores related to increasing age in adults over the age of 65, indicating that the difference between actual age and the age of 65 for adults older than 65 (adults under 64 had a value of 0) was included. In the models, the changes over time for adults over the age of 65 was assessed by the sum of two coefficients of age-45 and age-65. Second, to assess the difference based on the level of social activity in rate of change of cognitive performance over the years, the interactions of social activities with the variables of age-45 and the variable of age-65 were introduced. The decline (or increasing) rate in MMSE scores in adults over the age of 65 was assessed as the sum of between coefficients of two interaction terms. The present study analyzed the pooled sample and the sample stratified by gender, which resulted in three regression analytic models. In addition, we used the Wald test to examine whether the association of social activity with cognition depended on gender. Given that correlations between social activities were weak, ranging between 0.023 and 0.207, the association of four types of social activity with cognition was assessed in the same analytic model. All analyses were conducted with SAS 9.0 (SAS Institute Inc., Cary, NC, USA). The PROC MIXED procedure was used for multi-level linear mixed models.

## 3. Results

### 3.1. Descriptive Statistics

The baseline demographics of participants are depicted in Table 1. Of the total adults aged 45 years or older (n = 6706), 3347 (49.9%) were male and 3359 (50.1%) were female. The proportion of respondents who participated in religious meetings was 24.7%. The proportion of respondents who participated in social clubs or leisure, cultural, or sports groups was 62.6%. The proportion of respondents who participated in alumni societies, societies for people from the same hometown, or family councils was 25.8%. The proportion of respondents who participated in volunteer groups, political parties, non-government organizations, or interest groups was 4.6%.

### 3.2. Relationship between Social Activities and Cognitive Decline

Among all participants, MMSE scores decreased with increasing age, at an approximate rate of 0.18 per year between the ages of 45 and 65, as shown in all four columns (Table 2). The decrease in MMSE scores was steeper at up to 0.22 per year for adults aged 65+, indicating that MMSE scores decreased at an approximate rate of 0.39 per year over the age of 65. Baseline social activity (all kinds of social activity) was not related to the slope of changes of MMSE scores when age was less than 65, revealing insignificant coefficients of interaction terms between age-45 and each social activity. Baseline social gatherings (but not religious parties, alumni/clan gatherings, and volunteer work) were positively related to the slope of changes of MMSE scores when age was greater than 65 (*p* = 0.01). In other words, social gatherings were likely to delay the decline of cognitive function when individuals were older than 65, by approximately 0.08 units (Table 2).

Among men, MMSE scores decreased with increasing age, at a rate of approximately 0.18 units. The decrease in MMSE scores was steeper for those aged older than 65 compared with younger participants. At baseline, social activity of any kind was not related to the MMSE scores. However, baseline social activity did not appear to delay the decline of MMSE scores. Social activity may not impact the decline of cognitive function for men (Table 2).

**Figure 1:**
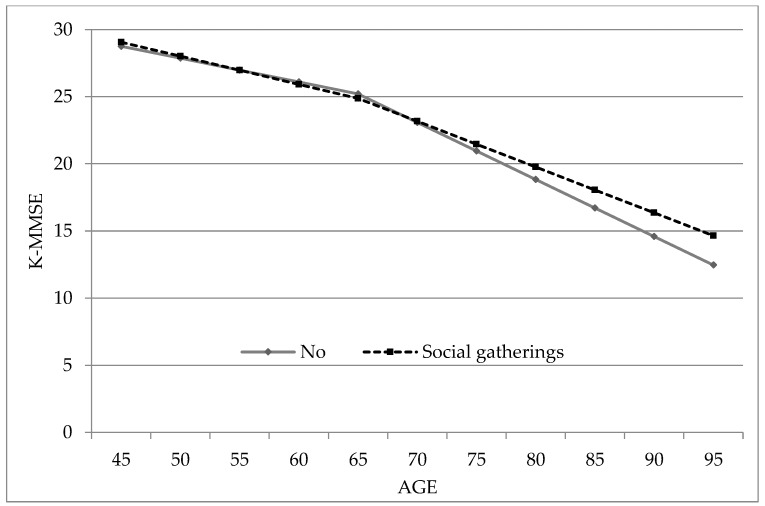
Change in K-MMSE over age based on participation in social gatherings among women.

Among women, MMSE scores decreased with increasing age at a rate of approximately 0.18 units. The decline in scores was steeper for those aged older than 65. At baseline, social activity of any type was not related to MMSE score. In addition, baseline social activity was not related to the decline in MMSE score in individuals younger than 65. Participation in social gatherings contributed to preserving the decline of MMSE scores (β = 0.118, *p* = 0.011), whereas religious activities, alumni/clan gatherings, and volunteer work did not exhibit significant relationships with the decline of MMSE scores in individuals older than 65. In other words, social gatherings were likely to delay the decline of cognitive function among women older than 65 (Table 2). Further, Wald tests on the interaction terms in the pooled samples revealed significant interaction effects based on gender (*p* = 0.027). Figure 1 presents the change in K-MMSE scores based on age among women who participated in social gatherings, which was estimated using the regression model.

## 4. Discussion

Using nationally representative longitudinal data for middle-aged and older Koreans, this study investigated the relationship between participation in different social activities and cognitive function by gender. The results indicate that social gatherings had a positive impact on preventing or delaying the decline of cognitive function, particularly among the older adults (over 65). One of the key findings is that the effect of social activities on cognitive function varied by gender, where women who participated in social gatherings were less likely to experience the decline of cognitive function when older than 65, whereas no effect of social gatherings on cognitive function was observed in men. Additionally, participation in social activities of any type had no impact on cognitive function in men, suggesting the need for a gender-stratified policy for preventing the decline of cognitive function while promoting engagement of social activities in the Korean older adults.

The findings from our study are consistent with previous studies suggesting that engaging in social activities in later life is more likely to prevent the decline of cognitive function at some level, and eventually reduce the risk of mental health problems and its related mortality [33,34,35]. Although the positive effects of greater participation in social activities on cognitive impairment in later life have mostly been observed, there are mixed findings on how different social activities impact cognitive function [8,17,34,36]. For instance, a previous study found that active volunteer work has a protective effect on cognitive function [36], whereas other studies suggested that active engagement in religious or social group activities contributes to preventing cognitive decline in both middle-aged and older adult populations [8,37,38]. In addition, a cross-sectional study examining the relationship between participation in social activities and MMSE decline among Korean middle-aged and older adult populations found that only religious and family-oriented activities were likely to delay cognitive function among individuals over 65, suggesting a community-based policy approach as one of the strategies to prevent and manage the increasing prevalence of dementia [37]. Studies from other Asian countries, such as China, Taiwan, and Japan, have also suggested that participation in activities associated with intellectually challenging activities and active interpersonal exchanges have positive effects on the prevention of cognitive decline regardless of biological aging [8,17,34]. Although the mechanism of how such activities decrease the risk of cognitive decline among the older adults is complex, ample evidence suggests that environments that provide social or cognitive interaction, resulting from greater participation in social activities, could be conducive to increasing cognitive reserve, thereby delaying the decline of cognitive function, regardless of biological aging [9,39]. Given the different nature of various social activities and cultures, one must be cautious in concluding that all types of social activities could be equally beneficial to cognitive function in the older adults.

Although there is no consensus on the possible mechanisms, there are possible explanations for the positive effects of social gatherings on cognitive decline in the Korean older adults. It has been suggested that psychological pathways might be involved in the beneficial effect on cognitive function [18]. Participation in friendship or leisure activity fulfills meaningful social roles, which could potentially sustain a person’s self-concept of worth and competence [40]. In this sense, our results, indicating social gatherings had beneficial effects on cognitive function, could be interpreted to mean that the older adults find socially meaningful roles or strengthen their self-efficacy through networking with friends and similar age group in community senior or social clubs.

In addition, older people who actively engage with social-tie activities might exhibit higher positive emotional states and less stressful daily life. Frequent interaction with close social ties generally leads to stress reduction and increased emotional stability, which is ultimately beneficial in preserving cognitive abilities compared with individuals with no interaction with others [41]. From a theoretical perspective, it is also possible that participation in social gatherings is associated with cognitive stimulations, contributing to the building of a cognitive reserve that ultimately optimizes its function [9,41]. For instance, activities involved with acquiring and organizing information, such as attending organization meetings and practicing an artistic or lecture activity, are associated with a reduced risk of dementia [9]. Therefore, continuous cognitive stimulation through involvement with such an activity might contribute to preserving cognitive decline, even enhancing cognitive reserve [9,41].

It is important to note why participation in other activities did not contribute to preserving cognitive decline. In fact, some studies suggest religious and volunteer activities also have positive relations with cognitive function. It is plausible that this conflicting result is attributed to the generally low levels of importance placed on religion in Korea compared with other countries, such as the U.S., where most studies on the relationship between religion and cognitive function were conducted. In Korea, religious activity is typically associated with personal belief and value, so religiosity may have smaller health effects than in countries where religious activity remains an important part of life [29,42]. Regarding volunteer work, the quality of volunteer work in Korea may not be sufficient to influence cognitive function for older-aged individuals [43]. Despite the increasing demand for volunteer work for older adults, systematic support and a formal compensation schemes are still lacking [29]. These limitations may hinder older adults in their attempt to engage more often in such activities and results in no effect on cognition. Several cross-sectional studies indicate that alumni and clan gathers are also beneficial to delaying cognitive decline; however, our results from a longitudinal analysis reveal no association. Most alumni and clan gatherings may be less cognitively demanding compared with other activities [44]. The frequency of social activity may also help to explain the conflicting results. For instance, participation in social gatherings (e.g., private saving or senior clubs), but alumni/clan gatherings are generally occasional events (e.g., semiannual or annual), which offer very limited influence on older adults’ cognitive function.

The effect of greater participation in such activities varied by gender. Although none of the social activities were associated with cognitive function, senior center-oriented activities were statistically associated with women only. Regarding the type of social participation, a previous study demonstrated that significant associations vary according to gender. Among females, participation in all social groups except for senior citizen clubs was inversely associated with a decline in efficacy, whereas among males, a beneficial effect on efficacy was restricted to hobby and volunteer groups [45]. Some studies have reported that social participation produces greater benefits for the health of males than females [46,47]. As they are more likely to achieve close relationships with their large and diverse networks compared with males, females tend to receive positive benefits from social participation, demonstrating that a constructive influence of social participation on efficacy is stronger for females compared with males [45]. One plausible explanation is that men are less likely to be involved in social interaction in later life given that marriage constitutes a large portion of their social life [48]. In contrast, women are often responsible for maintaining and fostering social activities after their counterparts retire and lose their social contact [49]. This given role in later life may lead women to participate in more activities, resulting in more benefits on cognitive function. Along with findings from previous studies, our study could help conclude that women of older ages need to be encouraged to be involved in certain types of social activities as part of healthy aging strategies. In addition, it is interesting to highlight that participation in social gatherings was effective to prevent cognitive function in older women, but not in younger women (under age 65). Because age is known to be a strong predictor of cognitive decline, it is possible that women under age 65 were less likely to experience cognitive decline compared to older women (over age 65). This helps explain why younger women may have no effect or limited effect of participation in social gatherings on cognitive preservation [17].

Our results indicate that social and health policy should be centered on encouraging social activities, including investing in physical and social infrastructure as part of older adults’ health policy intervention in terms of preventing cognitive decline and eventually preventing dementia. Although this study provides clear policy implications for the prevention of cognitive decline in Koreans older adults using a population-based longitudinal survey, there are some limitations that we have identified. The limitations of this study included the measures of social activity and health. Our measure of social activity did not contain information about the quality of these activities. In addition, data on health conditions were self-reported; therefore, it is possible that unmeasured health experiences potentially affected levels of memory and integration. We addressed this possibility by excluding baseline respondents with poor memory scores, but further studies with more intensive data collection on health status are required. We also identified the strengths of this study. Previous studies have linked social activities to lower cognitive abilities in later life [17,29,50]. These findings are mainly based on studies with a cross-sectional design or studies with short-term follow-up. These features limit the possibility of evaluating the longer-term associations between social activities and cognitive change and identifying some indication of the direction of the association, which results in excluding individuals interviewed at all data points, such as the deceased or dropouts, from the analysis. As these are the individuals most likely to experience cognitive impairment, this may lead to an overestimation of the beneficial impact of social activities. To overcome this identified limitation, our study adopted a longitudinal design with up to five measures of cognitive abilities over an eight-year period using a multi-level linear mixed model.

## 5. Conclusions

This study builds upon previous research by applying a more statistically sound approach, a multi-level linear mixed model, to examine how participation in social activities affects cognitive function in older Korean adults. This study found that MMSE scores decreased with increasing age, at a rate of approximately 0.18 units across all age-gender groups, and the decrease was steeper for adults aged 65 and over. Social gatherings were likely to delay the decline of cognitive function after the age of 65 by 0.079 units. In a gender-stratified model, the results demonstrated that social activity might not impact the decline of cognitive function in men. In women, participation in social gatherings was negatively related to the decline in MMSE scores, whereas baseline religious parties, alumni/clan gatherings, and volunteer work did not show significant relationships with the decline in MMSE scores. This finding could be interpreted as indicating that social gatherings are likely to delay the decline in cognitive function among women older than 65. This study suggests the need for a gender-stratified policy for preventing the decline in cognitive function while promoting engagement of social activities in the Korean older adults given that the effects of social gatherings on cognitive function differ based on gender.

## Figures and Tables

**Table 1:** Descriptive characteristics of the sample from the Korean Longitudinal Study of Aging (KLOSA).

Variables			All		Male		Female	
			Mean (SE) or %	n	Mean (SE) or %	n	Mean (SE) or %	n
			100	6706	49.9	3347	50.1	3359
Age			58.1 (0.12)		59.2 (0.17)		57.0 (0.16)	
Social activity	Religious parties	No	75.3	5047	82.1	2747	68.5	2300
		Yes	24.7	1659	17.9	600	31.5	1059
	Social gatherings	No	37.4	2510	35.3	1180	39.6	1330
		Yes	62.6	4196	64.7	2167	60.4	2029
	Alumni/clan gatherings	No	74.2	4974	65	2174	83.4	2800
		Yes	25.8	1732	35	1173	16.6	559
	Volunteer work	No	95.4	6397	95.5	3195	95.3	3202
		Yes	4.6	309	4.5	152	4.7	157
Marital status	Married		86.2	5781	92.9	3109	79.5	2672
	Single		13.8	925	7.1	238	20.5	687
Working status		No	52.1	3494	35.8	1199	68.3	2295
		Yes	47.9	3212	64.2	2148	31.7	1064
Residence area	Urban		79.5	5331	77.8	2605	81.2	2726
	Rural		20.5	1375	22.2	742	18.8	633
Education	Elementary school		7.6	508	4.9	163	10.3	345
	Middle school		25.3	1696	20	668	30.6	1028
	High school		52.9	3545	53.8	1802	51.9	1743
	College or above		14.3	957	21.3	714	7.2	243
Income	Q1		24.9	1669	23.4	782	26.4	887
	Q2		24.6	1651	24.9	833	24.4	818
	Q3		26.3	1766	27.2	909	25.5	857
	Q4		24.2	1620	24.6	823	23.7	797
Physical activity		Yes	44.4	2979	45.5	1524	43.3	1455
		No	55.6	3727	54.5	1823	56.7	1904
Drinking	Non-drinker		56.8	3808	34.6	1158	78.9	2650
	Current drinker		43.2	2898	65.4	2189	21.1	709
Smoking	Never smoked		67.5	4528	37.9	1267	97.1	3261
	Ever smoked		32.5	2178	62.1	2080	2.9	98
Activities of daily living (ADL)		None	99	6636	98.7	3305	99.2	3331
		1+	1	70	1.3	42	0.8	28
Depression		No	76.1	5101	79.4	2657	72.8	2444
		Yes	23.9	1605	20.6	690	27.2	915
Comorbidity		None	69.3	4649	68.5	2292	70.2	2357
		1+	30.7	2057	31.5	1055	29..8	1002
K-MMSE Score	2006		27.4 (0.02)	6706	27.5 (0.03)	3347	27.2 (0.03)	3359
	2008		25.7 (0.02)	5786	25.8 (0.10)	2876	25.7 (0.09)	2910
	2010		24.2 (0.02)	5363	24.3 (0.12)	2642	24.0 (0.12)	2722
	2012		25.1 (0.02)	5159	25.3 (0.13)	2530	25.0 (0.12)	2629
	2014		25.6 (0.02)	4725	26.0 (0.08)	2273	25.3 (0.09)	2452

**Table 2:** Relationship between social activity and cognitive performance from the Korean Longitudinal Study of Aging (KLOSA), 2006–2014 (n = 6706).

Variables *	All (n = 6706)			Male (n = 3359)			Female (n = 3347)		
	Coeff.	S.E.	*p*-Value	Coeff.	S.E.	*p*-Value	Coeff.	S.E.	*p*-Value
Age-45	−0.177	0.014	<0.001	−0.180	0.021	<0.001	−0.174	0.019	<0.001
Age-65	−0.219	0.026	<0.001	−0.169	0.035	<0.001	−0.270	0.038	<0.001
Religious parties (RP)	−0.010	0.179	0.957	−0.039	0.316	0.902	−0.044	0.218	0.841
(Age-45)·RP	−0.011	0.014	0.435	−0.015	0.023	0.507	−0.006	0.017	0.718
(Age-65)·RP	0.004	0.035	0.920	0.027	0.053	0.613	0.000	0.048	0.999
Social gatherings (SG)	0.292	0.162	0.072	0.288	0.256	0.260	0.305	0.208	0.143
(Age-45)·SG	−0.019	0.012	0.125	−0.006	0.019	0.772	−0.032	0.017	0.052
(Age-65)·SG	0.079	0.031	0.010	0.040	0.042	0.341	0.118	0.046	0.011
Alumni/clan gatherings (AG)	−0.157	0.167	0.346	−0.247	0.244	0.311	−0.039	0.243	0.874
(Age-45)·AG	0.001	0.013	0.966	0.008	0.019	0.681	−0.010	0.022	0.637
(Age-65)·AG	0.049	0.038	0.192	0.017	0.045	0.711	0.089	0.079	0.258
Volunteer work (VW)	0.172	0.334	0.607	0.082	0.521	0.876	0.188	0.433	0.665
(Age-45)·VW	0.016	0.027	0.556	0.024	0.040	0.556	0.014	0.037	0.703
(Age-65)·VW	0.023	0.084	0.782	−0.040	0.110	0.718	0.127	0.131	0.333
Sex	0.157	0.104	0.131	−	−	−	−	−	−
Age	0.174	0.010	<0.001	0.163	0.015	<0.001	0.180	0.014	<0.001
Marital Status	0.206	0.113	0.067	0.151	0.206	0.465	0.290	0.138	0.035
Work Status	0.001	0.080	0.992	−0.017	0.131	0.897	0.025	0.104	0.808
Residence (Rural)	0.156	0.088	0.076	0.132	0.127	0.297	0.180	0.123	0.144
Education-Elementary	−1.924	0.191	<0.001	−2.049	0.310	<0.001	−1.705	0.266	<0.001
Education-Middle school	−0.955	0.132	<0.001	−1.007	0.181	<0.001	−0.821	0.208	<0.001
Education-High school	−0.288	0.105	0.006	−0.407	0.132	0.002	−0.106	0.182	0.559
Income-Q1	−0.448	0.105	<0.001	−0.476	0.159	0.003	−0.420	0.140	0.003
Income-Q2	−0.319	0.102	0.002	−0.261	0.151	0.083	−0.363	0.140	0.010
Income-Q3	−0.014	0.094	0.882	−0.044	0.136	0.745	0.029	0.130	0.826
Physical Activity (No)	0.000	0.072	0.999	0.082	0.105	0.438	−0.093	0.100	0.353
Drinking (Current drinkers)	−0.024	0.079	0.760	0.016	0.111	0.882	−0.076	0.114	0.503
Smoking (Ever smoked)	−0.015	0.098	0.877	0.030	0.106	0.775	−0.519	0.293	0.077
ADL	−0.518	0.117	<0.001	−0.547	0.127	<0.001	−0.321	0.307	0.297
Depression (Yes)	−0.423	0.085	<0.001	−0.532	0.133	<0.001	−0.332	0.112	0.003
Comorbidity	−0.240	0.080	0.003	−0.269	0.114	0.018	−0.196	0.114	0.086

* All covariates except for Age-45 and Age-65 were measured at baseline. Dependent variable (K-MMSE), Age-45, and Age-65 were measured at each wave.
